# IgG serum levels in CVID patients during pregnancy

**DOI:** 10.1186/1939-4551-8-S1-A157

**Published:** 2015-04-08

**Authors:** Tatiane Pavan-Ramos, Ana Paula Willy-Fabro, Ana Carolina Rozalem, Juliana T Lessa Mazzucchelli, Beatriz Costa Carvalho

**Affiliations:** 1 Federal University of Sao Paulo, Brazil

## Background

Common Variable Immunodeficiency (CVID) is characterized by reduced serum levels of immunoglobulins (Ig) and its treatment requires regular infusion of intravenous immunoglobulin (IVIG). Eventually CVID female patients will reach reproductive age and it is known that maternofetal transplacental transport of antibodies is important to protect the child in the first months of life. The IgG present in fetal circulation comes from the mother after being actively transfered across the placenta. Although the required receptors for the transfer are expressed early on, most of it occurs in the third trimester of pregnancy and therefore this is the most important period for newborn protection. Transplacental transport of IgG is similar in pregnant women with and without CVID(Costa-Carvalho et al.).To this date, it is not established how the IVIG infusion should be conducted in order to maintain adequate IgG levels for the pregnant woman with CVID and her newborn.

## Objectives

To evaluate IVIG dose and serum levels of IgG during pregnancy in CVID patients.

## Methods

Retrospective analysis of chart data of pregnant CVID patients. All patients received IVIG every 4 weeks. Data on weight gain, IVIG doses received and their IgG serum levels obtained immediately before the monthly infusion, data on infectious episodes reported during visits, delivery and the newborns were collected.

## Results

Eight pregnancies in 6 patients were studied in CVID patients aged 20-37 years, all being either the first or the second pregnancy. They were using IVIG for an average of 3 years (± 2 years). All patients had pulmonary abnormalities as the main morbidity factor. Weight varied from a 2.2kg weight loss to a 11.6kg weight gain (mean 7.5kg ± 4.9kg). They received a mean IVIG dose of 648mg/kg/dose monthly. There was no difference between IgG levels before and during the pregnancies. There were an average of 1.5 infectious episodes reported during visits per pregnancy that required antibiotic use. All babies were full term, all had an average birth weight of 3,254g(±305g) and were discharged home 72 h after delivery.

## Conclusions

IVIG dose adjustment during pregnancy in CVID patients successfully maintained IgG levels without any disease aggravating factors.

